# Isolation and Characterization of the First Antigen-Specific EGFRvIII vNAR from Freshwater Stingray (*Potamotrygon* spp.) as a Drug Carrier in Glioblastoma Cancer Cells

**DOI:** 10.3390/ijms26030876

**Published:** 2025-01-21

**Authors:** Alejandro Manzanares-Guzmán, Andrea C. Alfonseca-Ladrón de Guevara, Elia Reza-Escobar, Mirna Burciaga-Flores, Alejandro Canales-Aguirre, Hugo Esquivel-Solís, Pavel H. Lugo-Fabres, Tanya A. Camacho-Villegas

**Affiliations:** 1 Unidad de Biotecnología Médica y Farmacéutica, Centro de Investigación y Asistencia en Tecnología y Diseño del Estado de Jalisco (CIATEJ), Guadalajara C.P. 44270, Jalisco, Mexico; almanzanares_al@ciatej.edu.mx (A.M.-G.); analfonseca_al@ciatej.edu.mx (A.C.A.-L.d.G.); eliarezza@gmail.com (E.R.-E.); acanales@ciatej.mx (A.C.-A.); hesquivel@ciatej.mx (H.E.-S.); 2 Centro de Nanociencias y Nanotecnología, Universidad Nacional Autónoma de México (CNyN-UNAM), Carretera Tijuana-Ensenada km107, Ensenada C.P. 22860, Baja California, Mexico; mirna.b.flores@ens.cnyn.unam.mx; 3 CONAHCYT-Unidad de Biotecnología Médica y Farmacéutica, Centro de Investigación y Asistencia en Tecnología y Diseño del Estado de Jalisco (CIATEJ), Guadalajara C.P. 44270, Jalisco, Mexico; plugo@ciatej.mx

**Keywords:** glioblastoma, EGFRvIII, variable new antigen receptor (vNAR), modelling and molecular dynamics simulation, targeted drug delivery, drug carrier, *Potamotrygon* spp.

## Abstract

Glioblastoma is the most common and highly malignant brain tumor in adults. New targeted therapeutic approaches are imperative. EGFRvIII has appealing therapeutic targets using monoclonal antibodies. Thus, endeavors toward developing new mAbs therapies for GBM capable of targeting the tumor EGFRvIII biomarker must prevail to improve the patient’s prognosis. Here, we isolated and characterized an anti-EGFRvIII vNAR from a non-immune freshwater stingray mixed library, termed vNAR R426. The vNAR R426 and pEGFRvIII interaction was demonstrated by molecular docking and molecular dynamics, and the recognition of EGFRvIII in vitro was further confirmed by cell immunofluorescence staining. Moreover, the vNAR R426 was shown to be an effective cisplatin drug carrier in the U87-MG glioma cell line. The cisplatin-coupled vNAR demonstrated highly significant differences when compared to free CDDP at 72 h. Notably, the cisplatin-vNAR carrier achieved better efficacy in the U87-MG cell line. Thus, we described the vNAR R426 internalization by receptor-mediated endocytosis and the subsequent COPI-mediated nuclear translocation of EGFRvIII and highlighted the importance of this shuttle mechanism to enhance the targeted delivery of cisplatin within the glioma cell’s nucleus and improved cytotoxic effect. In conclusion, vNAR R426 could be a potential therapeutic carrier for EGFRvIII-targeted glioblastoma and cancer therapies.

## 1. Introduction

Glioblastoma (GBM), a highly malignant brain tumor, is a grade IV astrocytoma [1]. Multimodal treatment involves maximal safe surgical resection, chemotherapy, and radiotherapy. Still, GBM patients experience tumor progression and poor prognosis [2,3,4,5,6,7,8,9,10]. Cisplatin (CDDP) is a cytostatic and DNA-damaging drug widely used in first-line systemic chemotherapy against epithelial malignancies and second- and third-line treatment strategies against metastatic malignancies, including GBM [11]. The epidermal growth factor receptor (EGFR) has been reported to be upregulated in several cancers, including glioblastoma [12,13,14,15,16,17]. Gene EGFR overexpression has been observed in 50% of GBM cases [14], and EGFRvIII, the prevalent EGFR mutation, was found in 25–33% of all GBM patients. EGFRvIII is only found in malignant cells and develops from the genomic deletion of exons 2–7, disrupting the extracellular domain while retaining intact transmembrane and intracellular kinase domains [18,19,20,21]. Thus, EGFRvIII shows ligand-independent signaling and is constitutively active [14]. EGFRvIII is a well-established target for GBM therapeutic development, providing the potential for specificity in combination with efficacy and safety [22]. During therapy with mAbs targeting the native EGFR, dermal toxicities have been commonly observed [22]. Thus, targeting the tumor-restricted mutant EGFRvIII should not prompt these associated adverse effects.

Single-domain antibodies (sdAbs) are part of the heavy chain-only antibodies. They are the autonomous variable single domains, termed variable fragments, of heavy chain antibodies (VHH) in camelids and the shark variable New Antigen Receptor (vNAR) in Elasmobranchs [23,24,25]. vNAR is the smallest antibody domain in the animal kingdom (12–15 kDa) that can effectively target antigens [26,27]. vNAR possesses unique characteristics such as high stability, solubility, affinity, recombinant expression (in procaryotic or eukaryotic systems), and the potential to be modified [23,28,29,30,31]. Contrastingly, conventional mAbs efficacy is hindered due to their molecular weight (~150 kDa) and intrinsic physical complexity, including poor stability and aggregation proneness [26,32,33,34]. vNAR proficiency has been extensively reported in the biomedical field as a potential diagnostic and therapeutic tool [35,36,37,38,39,40,41,42], including in brain diseases, as vNAR is capable of targeting and binding cryptic peptides, and its proficiency for blood–brain barrier passage to reach intracerebral targets has been also demonstrated [43,44,45].

To isolate target-specific sdAbs using display technology, the construction of a suitable antibody library is essential. Numerous types of vNAR libraries have been generated, including immune libraries, non-immune libraries (also termed naïve libraries), synthetic libraries, or semi-synthetic libraries [31,42]. Several studies have successfully isolated vNAR from naïve libraries in *G. cirratum* [42]. As an unbiased resource for antigen-specific vNAR isolation, diverse applications include liver cancer, Her2+ tumors, solid tumors, MERS, and SARS virus, and *Pseudomonas* infection [42]. Remarkably, these sdAbs maintained their selectivity, recognition ability, and high affinity toward their molecular targets screened by the phage display technique [42]. Moreover, naïve libraries can be generated from animals that cannot be actively immunized for ethical reasons and can produce antibodies faster than immune libraries [46].

Currently, a few studies have reported vNAR for GBM treatment [47]. Interestingly, an anti-EGFRvIII vNAR isolated from a freshwater stingray library has not been previously reported. *Potamotrygonidae*, including stingrays, are the only group of Elasmobranchs reported to have exclusively speciated in freshwater [48].

In the present study, we report the isolation, characterization, and evaluation of a vNAR anti-EGFRvIII (vNAR R426) from a non-immune mixed library of *Potamotrygon* (spp.), which proved in vitro and in silico EGFRvIII recognition. Here, we demonstrated the vNAR R426 internalization by receptor-mediated endocytosis and subsequent nuclear translocation of the vNAR R426-EGFRvIII complex mediated by COPI, and validated vNAR R426 as a cisplatin drug carrier in the glioblastoma U87-MG cell line. Our study provides a novel design and experimental basis for developing forthcoming systems for targeted glioblastoma therapy.

## 2. Results

### 2.1. Biopanning of a Phage-Display Library from Freshwater Stingrays

**Figure 1:**
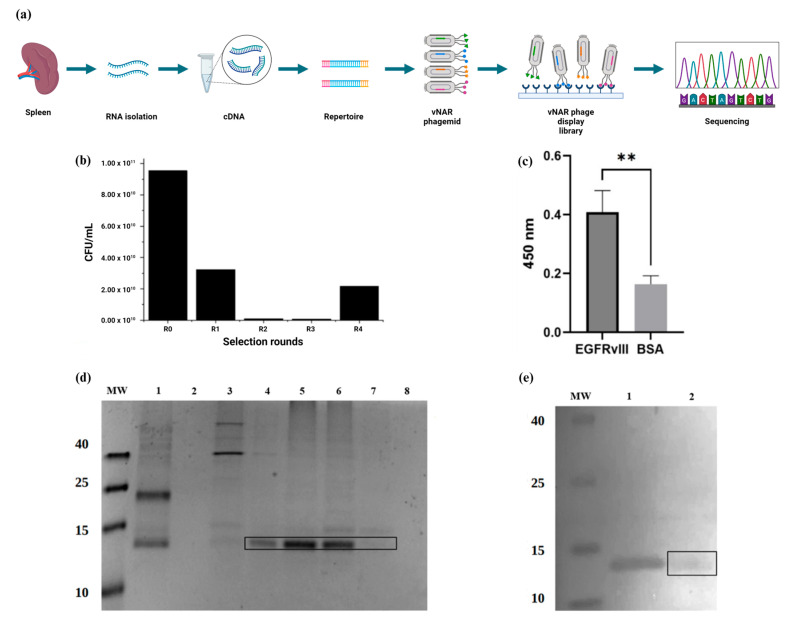
Identification of freshwater stingray-derived anti-EGFRvIII vNAR. (**a**) Schematic representation of non-immune phage library construction from freshwater stingray and sequencing. (**b**) Panning rounds with non-immune vNAR library. R = Round, CFU = Colony-forming units. (**c**) Recognition ELISA for vNAR R426-EGFRvIII peptide (LEEKKGNYVVTDHC). The hatched bars depict specific recognition of vNAR R426 towards the EGFRvIII receptor. Negative control: BSA 3%. Student’s *t* test = ** greatest significant difference with *p* < 0.01. (**d**) SDS-PAGE showing the purification steps of vNAR R426 in a 15% SDS-PAGE gel stained with Coomassie blue. MW = Molecular weight marker. 1 = non-retained fraction (NR), 2 = Negative control (C−) *E. coli* BL21 extract without plasmid, 3 = Wash 1 (50 mM imidazole), 4 = Elution 1 (E1), 5 = Elution 2 (E2), 6 = Elution 3 (E3), 7 = Elution 4 (E4), 8 = Elution 8 (E4). (**e**) Western blot of vNAR R426, MW = Molecular weight marker, 1 = Positive control (a non-related vNAR with 6xHis-Tag), 2 = Purified and endotoxin-free vNAR R426. Black boxes indicate the presence of vNAR R426 (~14.9 kDa).

A non-immune mixed library was generated using total RNA extracted from *Potamotrygon* spp. and pCOMb3X phagemid following a previous method [39]. For the isolation of multiple phages displaying a diverse library of anti-EGFRvIII specific vNAR domains (Figure 1a), the phage display was performed by four rounds of biopanning, culminating in final titers of 2 × 10^10^ CFU/mL (Figure 1b). Then, PCR was used to analyze isolated clones using specific primers; only positive clones were sequenced, as previously described [39].

**Figure 2:**
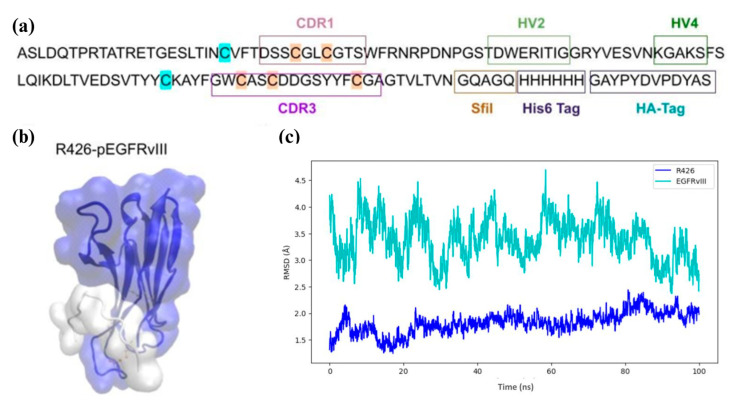
Molecular dynamics of the interaction between vNAR R426 and EGFRvIII peptide. (**a**) vNAR R426 sequence. CDR = Complementarity-determining region 1 and 3 (CDR1 and CDR3); HV = Hypervariable regions 2 and 4 (HV2 and HV4) are indicated in the box. Canonical cysteine residues are highlighted in blue and non-canonical cysteine residues are highlighted in orange. (**b**) Molecular representation of vNAR R426/pEGFRvIII complex. (**c**) Root Mean Square Deviation (RMSD) of vNAR and pEGFRvIII in the complex over 100 ns of molecular dynamics. The RMSD of vNAR R426 (Cα atoms) is blue. The RMSD of EGFRvIII peptide (Cα atoms) are in cyan. On average, there are 3 experimental replicates.

The final selection of vNAR clones that express and recognize the pEGFRvIII was confirmed by an ELISA assay (Figure 1c). The successful selection of the vNAR named R426 (113 amino acid residues) and subcloning into the pET28a+ plasmid using the NcoI and NotI sites (Figure 2a) to obtain the recombinant plasmid (pET28a+/vNAR R426) was achieved. The vNAR R426 gene sequence was subsequently employed for the following in silico characterization, and the recombinant plasmid enabled the recombinant vNAR protein production, followed by purification using Ni-NTA chromatography and Western blot (Figure 1d,e).

### 2.2. Selection of Appropriate Quality vNAR-R426 Structure

The next step in the in silico study was the selection of quality vNAR R426 structures for docking simulation studies. The structure evaluation before and after molecular dynamics is significant in determining the best energy-optimized structure for subsequent docking and simulation assays to make the best possible predictions. The energy of the post-molecular dynamics vNAR R426 structure achieved a minimal energy state as it has 93.7% residues in the favored region as opposed to 91.0% in the pre-molecular dynamics vNAR R426 of the Ramachandran plot. Moreover, 97.3% of residues (pre-molecular dynamics) and 99.1% (after molecular dynamics) were plotted and allowed for regions and disallowed of the Ramachandran plot.

### 2.3. In Silico Characterization of the Interaction Between vNAR R426 and EGFRvIII Peptide by Molecular Docking and Molecular Dynamics

Following the modeling of the vNAR R426 structure, high-quality vNAR R426 structures were selected for docking simulations. Molecular docking and interaction analyses revealed the specific contacts between vNAR R426 and the EGFRvIII peptide (pEGFRvIII), highlighting the critical role of the CDR3 region in target recognition (Figure 2b).

Molecular dynamics simulations demonstrated the conformational stability of complex vNAR R426-pEGFRvIII (Figure 2c). The system was subjected to a 100 ns MD simulation, and its stability was evaluated by tracking the deviations of all Cα atoms throughout the simulation (Figure 2c). The binding free energy (ΔG) of the complex was calculated to be −21.247 kcal/mol (Table S1) and physicochemical properties of vNAR R426 were determined (Table S2).

### 2.4. vNAR R426 Demonstrated Internalization by Receptor-Mediated Endocytosis

**Figure 3:**
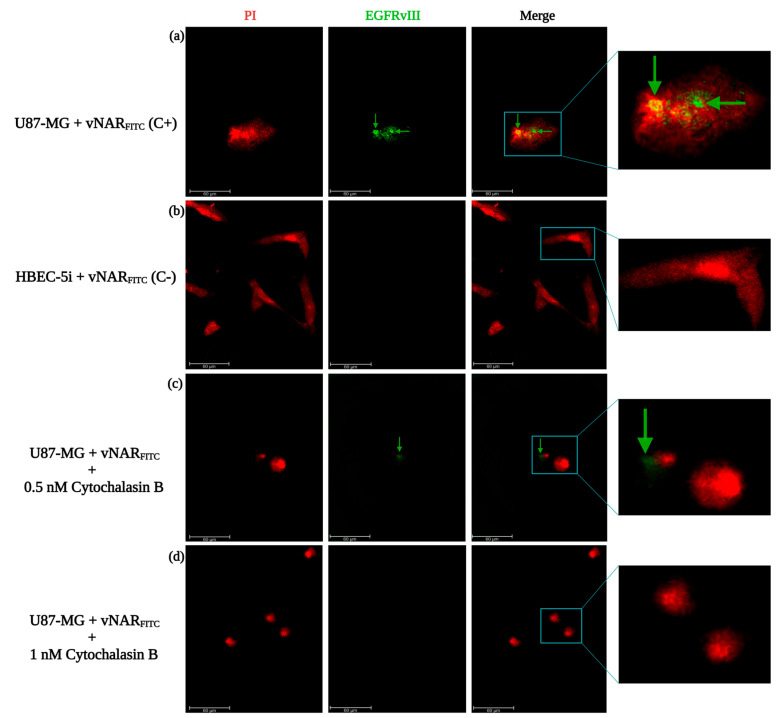
Immunofluorescence evaluation of vNAR_FITC_ binding to EGFRvIII and cell internalization. (**a**) U87-MG (EGFRvIII^+^ cells) as positive control (C+) and (**b**) HBEC-5i (wtEGFR^−^/EGFRvIII^−^ cells) as negative control (C−), were incubated for 4 h with vNAR_FITC_ (0.081 µM, final concentration), and treated with cytochalasin B at 0.5 nM (**c**) or 1 nM (**d**). vNAR_FITC_ signal (in green, as remarked by green arrows) and nucleus staining with propidium iodide (red). Scale bar, 60 µm. All experiments were conducted in triplicate.

First, we conjugated the vNAR R426 with Fluorescein-5-isothiocyanate (FITC), termed vNAR_FITC_; then, we incubated the U87-MG cell line (EGFRvIII^+^/wtEGFR^+^) and the cerebral microvascular endothelial cell line HBEC-5i (EGFRvIII^−^/wtEGFR^−^) with vNAR_FITC_ (Figure 3). Cytochalasin B, a vesicle formation inhibitor by actin filament function blockade, was employed to impede receptor-mediated endocytosis [49]. As seen in the immunofluorescence images, the vNAR_FITC_ signal was detected in U87-MG cells as EGFRvIII^+^ (Figure 3a,c). Nevertheless, no signal was detected in HBEC-5i cells (Figure 3b). The vNAR_FITC_ was localized in the cell membrane and nucleus of U87-MG cells (Figure 3a). Moreover, vNAR_FITC_ binding was achieved after 4 h in U87-MG cells (Figure 3a–d), and cytochalasin B treatment effectively inhibited vNAR_FITC_ internalization by receptor-mediated endocytosis in U87-MG cells (Figure 3d).

### 2.5. vNAR R426 Can Be Carried Alongside EGFRvIII for Nuclear Translocation

**Figure 4:**
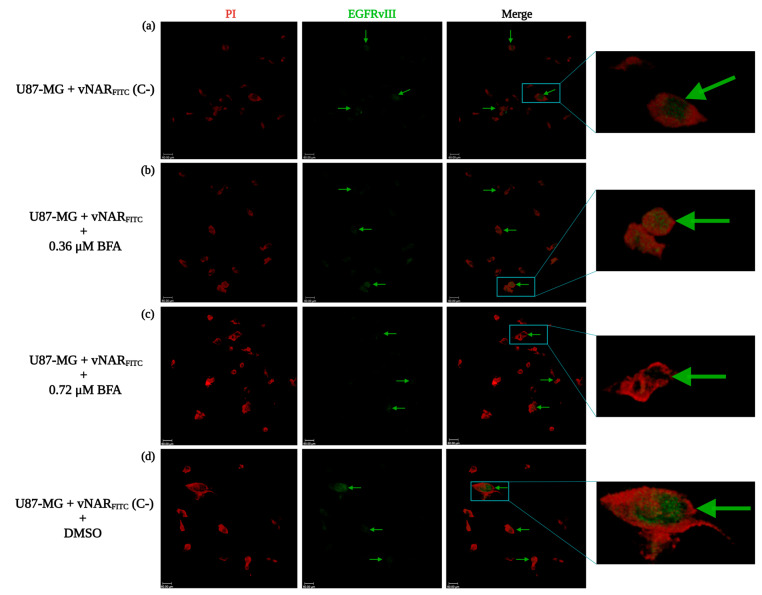
Inhibition of EGFRvIII nuclear translocation hampers vNAR_FITC_ signal within the cell nucleus. U87-MG cells were treated with or without DMSO or different concentrations of BFA for 30 min. Next, all cell groups were incubated with vNAR_FITC_ (0.081 µM) for 24 h and then immunofluorescence detected with confocal microscopy. (**a**) Negative control group (C−). (**b**) BFA treatment group at 0.36 µM. (**c**) BFA treatment group at 0.72 µM. (**d**) DMSO control group (C−). DMSO is the control of BFA. vNAR_FITC_ signal (in green, marked by green arrows). Nucleus staining with propidium iodide (red). Scale bar, 60 µm.

To establish the mechanistic basis of vNAR_FITC_ binding and nuclear translocation alongside EGFRvIII, we treated U87-MG with brefeldin-A (BFA), an inhibitor of the shuttle function of COPI vesicles. BFA inhibits intracellular COPI-mediated transport from Golgi to endoplasmic reticulum (ER), culminating in EGFRvIII nuclear translocation blockade [50]. Based on receptor-mediated endocytosis by vNAR R426 and COPI-mediated EGFRvIII nuclear translocation, we asked whether BFA can inhibit EGFRvIII nuclear translocation and the subsequent vNAR_FITC_ signal within the nucleus. U87-MG cells were incubated with vNAR_FITC_ and BFA groups were also treated with increasing concentrations of BFA. After 24 h of incubation, vNAR_FITC_ was in the nucleus of the negative control groups (Figure 4a,d). BFA treatment cells decreased the amount of fluorescent signal within the nucleus in a concentration-dependent manner (Figure 4b,c) when compared to the control group (vNAR_FITC_ vs. control group). These results were consistent with a previous study that hampered COPI-mediated EGFRvIII nuclear translocation via BFA treatment in U251 cells [50]. These findings imply that vNAR_FITC_ nuclear translocation relies on EGFRvIII nuclear translocation mediated by the COPI vesicle, establishing the mechanistic basis for vNAR_FITC_. Moreover, the inhibition of this mechanism also hinders vNAR_FITC_ nuclear translocation.

### 2.6. vNAR R426 Can Deliver Cisplatin to U87-MG Cells with High Efficiency

**Figure 5:**
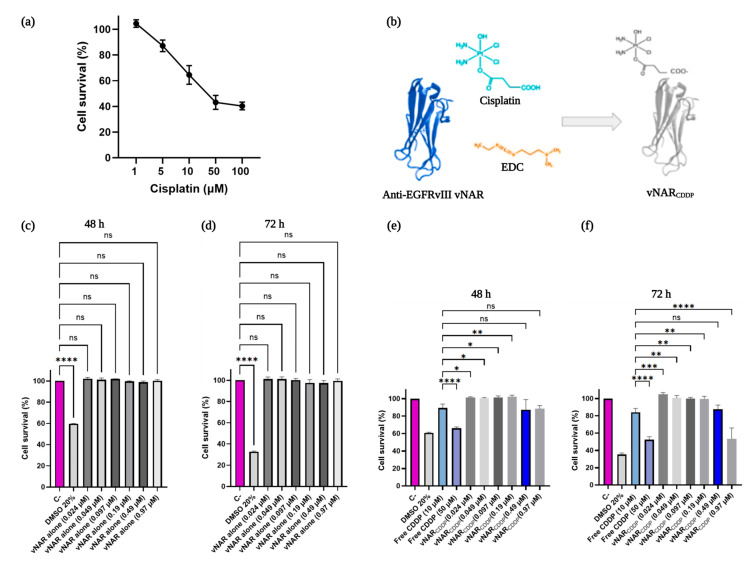
(**a**) Cell viability assay of U87-MG cells incubated with cisplatin. U87-MG cells were incubated with different concentrations of cisplatin (CDDP) for 72 h. The means for each concentration are represented. The experiments were performed in triplicate. (**b**) Schematic representation of vNAR R426 and cisplatin conjugation. The anti-EGFRvIII vNAR was coupled with cisplatin following the EDC coupling protocol. The cisplatin-vNAR R426 carrier (vNAR_CDDP_) was obtained. Anti-EGFRvIII vNAR (Blue), cisplatin (Cyan), EDC (Orange), and vNAR_CDDP_ (White) are depicted. (**c**,**d**) Cell viability assay of vNAR R426 alone and cisplatin-vNAR R426 carrier in U87-MG cells. U87-MG cells were incubated with different concentrations of vNAR R426 alone for 48 h (**c**) and 72 h (**d**). (**e**,**f**) U87-MG cells were incubated with varying concentrations of cisplatin-vNAR R426 carrier (vNAR_CDDP_) for 48 h (**e**) and 72 h (**f**). Data are presented as mean ± SD. The experiments were performed in triplicate. One-way ANOVA for the comparison of the free CDDP treatment (10 μM) to the means of the other therapies, considering *p* < 0.05 significant, ns > 0.05, * *p* < 0.05, ** *p* < 0.01, *** *p* < 0.001, **** *p* < 0.0001.

First, we performed a cell viability assay with different concentrations of cisplatin (1 µM–100 µM) in U87-MG cells (Figure 5a). To demonstrate the targeted delivery of cisplatin via EGFRvIII-mediated endocytosis, vNAR R426 was coupled with cisplatin (Figure 5b). We subsequently assessed U87-MG cell survival with different concentrations of vNAR alone (0.024 µM–0.97 µM); non-significant differences were found with the treated groups compared to the negative control (cells without vNAR nor CDDP) at 48 h and 72 h (Figure 5c,d). Finally, we assayed the cisplatin-vNAR R426 carrier (vNAR_CDDP_) with different concentrations (0.024 µM–0.97 µM). In these cell viability assays, the concentration of vNAR R426 was normalized using the same concentrations of vNAR_CDDP_ as in the cell survival assay with vNAR alone. Thus, we further evaluated vNAR_CDDP_ in U87-MG cells at 48 h and 72 h (Figure 5e,f). The vNAR_CDDP_ (0.97 µM) demonstrated highly significant differences (~30% cell dead) when compared to the concentration of free CDDP (10 µM) at 72 h (Figure 5f). It is noteworthy that vNAR_CDDP_ (0.97 µM), which is ten-fold lower than the concentration of free CDDP (10 µM), achieved greater efficacy.

Based on the data, the IC_50_ of cisplatin in U87-MG cells was determined to be close to 39.2 µM. Nevertheless, as seen in Figure 5a, 10 µM of free CDDP results in ~30% cell death (70% survival) after 72 h of treatment, whereas in Figure 5f, the same concentration of free CDDP causes less than 20% cell death after 72 h of treatment. These apparent inconsistencies in IC_50_ variations are partly due to differences in initial cell density and the proliferative potential of the cell line [51]. Other methods like apoptosis analysis or cell counting do not correct these variations, indicating that intrinsic resistance to treatments is an inherent property of cancer cells. Moreover, a study revealed similar findings with the MTT assay, as it rarely yields consistent IC_50_ values against a specific cancer cell line for a given chemical compound. In summary, IC_50_ values are inherently variable due to the natural characteristics of cancer cells, and it is crucial to adopt a dynamic and more rigorous approach for evaluating drug resistance in both research and clinical studies [51]. However, we found consistency regarding the cell viability results of 10 µM of free CDDP in the U87-MG cell line compared to the negative control (cells only) [52].

**Figure 6:**
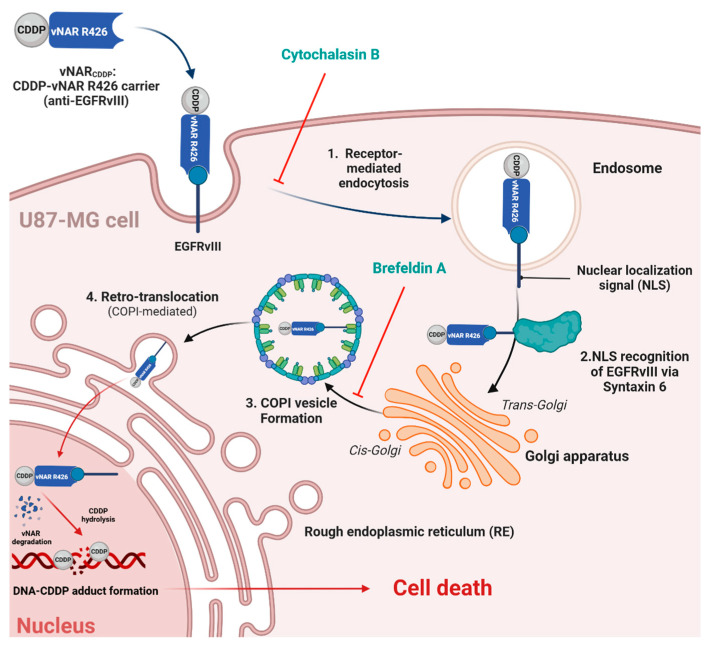
Schematic representation of the hypothetical vNAR_CDDP_ cytotoxic mechanism in U87-MG cells. The vNAR_CDDP_ recognizes the EGFRvIII receptor in the plasma membrane of U87-MG cells. Then, receptor-mediated endocytosis and endosome formation take place (**1**). The nuclear localization signal (NLS) within the EGFRvIII is recognized by syntaxin 6, which transports the vNAR_CDDP_-EGFRvIII complex to trans-Golgi (**2**). Next, COPI vesicle formation occurs in cis-Golgi and mediates the retro-translocation to the rough endoplasmic reticulum (**3**). Finally, vNAR_CDDP_ reaches the cell nucleus along with EGFRvIII, degrades, and CDDP can form adducts with DNA, culminating in cell death (**4**). The previously reported COPI-mediated EGFRvIII nuclear translocation [50] helped us hypothesize the vNAR_CDDP_ cytotoxic mechanism within the U87-MG cells. Red lines depict the inhibition checkpoints we employed in the cell assays.

Since cisplatin affects the cell cycle associated with the formation of CDDP-DNA adducts within the nucleus, COPI-mediated EGFRvIII-vNAR_CDDP_ complex retro-translocation to the nucleus can improve the CDDP-DNA adduct formation within the nucleus, translating in the compelling cytotoxic effect observed in U87-MG tumor cells (Figure 5f). vNAR R426 may reach the nucleus through its co-transport with EGFRvIII, as previously demonstrated (Figure 4). As such, the COPI-mediated EGFRvIII nuclear translocation could also serve as a shuttle mechanism for vNAR R426 (Figure 6).

## 3. Discussion

One of the major drawbacks for improving GBM treatment is the low diffusion of therapeutic agents across the blood–brain barrier (BBB) [9]. Because of their large size (150 kDa), the diffusion of conventional antibodies is hampered in tissues, and the employment of these for brain diseases, including glioblastoma, is highly limited by the BBB, as the amount of the peripherally administered antibodies capable of reaching the brain parenchyma is less than 0.1% [44,53]. On the other hand, vNARs could prevail over these limitations by virtue of their smaller size (12–15 kDa), which translates into their unique pharmacokinetic properties [44]. Moreover, vNAR is part of the Ig superfamily, and its structure is arranged in a distinctive β-sandwich fold, comprised by eight β-strands, resulting from the deletion of the framework 2 (FR2)-CDR2 region. On the other hand, the mammalian V domains are integrated by ten β-strands. Additionally, vNAR only holds two regions of high variability, the complementarity-determining regions 1 and 3 (CDR1 and CDR3). Contrarily, the mammalian variable region (V_H_ or V_L_) retains three CDRs each [23,26]. CDR3 is the most diverse region of vNAR. Upon antigen recognition, somatic mutation takes place in vNAR, as revealed in CDR1, a truncated CDR2 site, and TCR–HV4 loop regions. HV2 and HV4 depict these mutation-prone regions [23,35]. In contrast to the antigen-binding affinity of traditional antibodies, which relies on the six loops contained in two chains, vNARs have shown a superior antigen-binding affinity, owing to its four antigenic-binding loops (CDR1, CDR3, HV2, and HV4) [23,36,37]. Furthermore, the vNAR binding affinities through its CDR3 region have been reported in the nanomolar range toward an antigen [36,38]. Notably, the lowest binding affinity reported for an anti-HSA vNAR was in the picomolar range [37]. Previously, we reported the successful isolation of the first pan-specific single-domain vNAR (vNAR T1) capable of targeting the human TGF-β isoforms (β1, β2, and β3). vNAR T1 was isolated from a non-immunized *Heterodontus francisci* shark library, selected through phage display [54]. Notably, vNAR T1 showed an affinity (KD) of 9.61 × 10^−8^ M and binding for the same amino-acid as TβRI and TβRII cytokine receptors [54,55,56], supporting the potential of non-immune libraries to select biotechnologically relevant vNARs.

Clinically approved anti-EGFR mAbs have been broadly employed as a treatment for several types of cancer [57,58]. Nevertheless, the therapeutical inhibition of EGFR efforts in GBM patients, even at high intratumoral drug concentrations, have been primarily fruitless [57,59,60]. Most clinical trials in GBM are focused on cetuximab and nimotuzumab, both having discouraging results in clinical phase II and III trials, respectively [57,61,62,63]. Cetuximab demonstrated these disappointing outcomes due to its low BBB diffusion, limiting its capacity to reach the tumor [62,64].

Transferrin receptor 1 (TfR1)-mediated transcytosis is an efficacious approach to deliver protein therapeutics into the brain. A high affinity vNAR (TXB2)-targeting TfR1 has demonstrated the expeditious and successful crossing of the BBB and delivery of protein cargo to the brain, with equal affinity to human and murine TfR1 [23,53]. TXB2-hFc exhibited significant brain uptake in vivo by TfR1 transport mechanisms. Thus, TXB2 has been employed as an effective carrier for a broad diversity of biotherapeutics from the blood to the brain [53]. TXB2’s fusion with Bapineuzumab, a humanized anti–amyloid-beta mAb, demonstrated the capacity to reach the brain. Hence, the effective brain delivery of an IgG antibody was achieved by its fusion to an anti-TfR1 vNAR (TXB2). Notably, the concentrations of Bapi-TXB2 were threefold higher than those in Bapineuzumab within the brain. The brain-to-blood concentration ratio increased over time due to interactions with intracerebral Aβ deposits in transgenic mice overexpressing human Aβ and was observed for up to 6 days after the injection [53]. Several patents have been approved for vNAR with the capacity to cross the BBB [65,66,67,68]. Recently, a study assessed anti-IL-13Rα2 vNARs from an immune library from *Chiloscyllium plagiosum* (whitespotted bamboo shark) in A172 glioma cells. The results indicated that these vNARs had a significant capability to inhibit both the growth and migration of high IL-13Rα2 expressive glioma cells by hindering IL-13Rα2 on the cell surface [47]. These findings suggest that vNAR may render an effective delivery system for anti-GBM treatment.

A recent study tested the antitumor activity and mechanisms involved in the binding and blocking of EGFRvIII signaling of all clinically approved anti-EGFR mAbs and chimeric mAb 806 (ch806) towards a panel of gliomaspheres derived from patients and an intracranial orthotopic model [57]. The anti-EGFR mAbs, targeting native EGFR domain III, such as cetuximab, necitumumab, nimotuzumab, and matuzumab, did not neutralize EGFRvIII activation. While chimeric mAb 806 (ch806) neutralized EGFRvIII, it did not neutralize the native EGFR activation. The only reported anti-EGFR mAb capable of neutralizing both EGFRvIII and EGFR was panitumumab, with exceptional antitumor activity in vitro and in vivo [57]. Panitumumab prompted EGFRvIII internalization and recycling backward to the cell surface by firmly crosslinking the receptor, preventing activation. However, when panitumumab is internalized, it appears not degraded but shuttled back to the cell surface, consistent with receptor recycling [57]. Despite panitumumab efficacy towards EGFRvIII neutralization, the EGFR neutralization may represent a drawback, considering that native EGFR is also expressed in several healthy cell types [12,57]. Thus, endeavors toward developing anti-EGFR therapies for GBM capable of specifically targeting the tumor-specific EGFRvIII must prevail to prevent native receptor recognition.

In the present study, we isolated a single domain anti-EGFRvIII vNAR (vNAR R426) from a non-immune *Potamotrygon* (spp.) mixed library (Figure 1). We successfully characterized the interaction of vNAR and pEGFRvIII by molecular docking and molecular dynamics (Figure 2). The vNAR R426 demonstrated in vitro cell internalization by receptor-mediated endocytosis (Figure 3) and allowed translocation to the cell nucleus via the COPI-mediated EGFRvIII retro-translocation mechanism (Figure 4 and Figure 6). The vNAR_CDDP_ (0.97 µM) concentration demonstrated ~30% less cell survival compared to free CDDP (10 µM) at 72 h (Figure 5f), showing that the vNAR R426 was more efficient than free CDDP and has potential as an immunocarrier.

Conventionally, only 1% of the administered cisplatin reaches its target within cells [69]. The cell uptake of cisplatin occurs through the copper transporter (CTR1); after cisplatin uptake, the rapid degradation of CTR1, as previously demonstrated in human cells, prompts resistance to cisplatin due to its decreased influx [70]. EGFR nuclear translocation has been reported in several types of cancer, and nuclear EGFR signaling is involved in DNA repair, tumor progression, and cell proliferation [71,72,73,74,75]. Thus, nuclear EGFR (nEGFR) accumulation is a potential therapeutic target that rarely occurs outside the context of cancer [76], and full-length EGFR seems to translocate to the nucleus [62]. The translocation of the nEGFR leads to conversion from lysosomal degradation to nuclear trafficking [76]. The nuclear translocation of wtEGFR/EGFRvIII is mediated by the nuclear localization signal (NLS) within the intracellular domain; NLS allows interaction with importin-β, and the subsequent binding of the nucleoporins found in the nuclear pore complexes [62,77]. Moreover, wtEGFR/EGFRvIII nuclear translocation and binding with DNA-dependent protein kinase (DNA-PK) have been reported following cisplatin treatment [78]. Thus, the cell uptake of cisplatin via the vNAR R426 carrier (vNAR_CDDP_) could be more effective, since vNAR_CDDP_ targets the tumor-restricted EGFRvIII instead of CTR1, which generally depicts a cisplatin resistance mechanism by EGFRvIII nuclear translocation [78]. A drug delivery system by an anti-EGFR nanobody (7D12-9G8) for EGFR^+^ tumor cells has been reported [79]. Consistently, nanobody-drug conjugate selectively eliminates EGFR^+^ cancer cells, demonstrating a higher CDDP accumulation in cells via EGFR-mediated endocytosis than free cisplatin. However, the details of the drug carrier internalization, CDDP hydrolysis from it, and interaction within the nucleus are not declared [79]. Several studies have reported sdAbs for intracellular applications capable of reaching the cell nucleus [80,81,82,83]. Nevertheless, neither the interaction within the nucleus nor the nuclear degradation of the reported nanobodies were fully disclosed.

This study provides an anti-EGFRvIII vNAR and lays out the initial proof-of-concept experiments that support vNAR R426 as an effective drug carrier for receptor-mediated endocytosis. Our findings proved the mechanistic basis for the retrograde trafficking of the EGFRvIII-vNAR_CDDP_ complex and the compelling cytotoxic effect observed in glioma cells. The ubiquitin–proteasome system (UPS) is the most recurrent mechanism for protein quality control and degradation within eukaryotic cells [84]. Once vNAR_CDDP_ reaches the nucleus via the retrograde trafficking of EGFRvIII, vNAR_CDDP_ degradation could occur via the ubiquitin–proteasome system [84,85]. CDDP hydrolyzes from the vNAR_CDDP_ carrier, leading to DNA-CDDP adduct formation, culminating in glioma cell death. Nevertheless, this approach needs to be further examined. More information regarding sdAb fate within the nucleus could enhance our understanding and improve the design of forthcoming systems with nuclear targets.

## 4. Materials and Methods

### 4.1. Generation of a Non-Immune Library from Freshwater Stingrays

#### 4.1.1. Immune Library Amplification

A non-immune mixed library was generated using total RNA extracted from *Potamotrygon* spp. and pCOMb3X phagemid following a previous method [39]. A total of 5 µL of the ligation reaction was added to 100 μL of electrocompetent *E. coli* TG1 cells (200123, Agilent, Santa Clara, CA, USA), then electroporated and resuspended in SOC medium, and incubated at 37 °C, 250 rpm for 1 h. A series of incubations, with media addition and antibiotics within the recovered cells, were performed according to a previous protocol [39]. Then, cell culture was transferred to a sterile flask containing SB medium (198 mL) supplemented with ampicillin (100 µg/µL) and a helper phage M13K07 (2 mL; N0315S, New England Biolabs, Ipswich, MA, USA). The culture was incubated for 2 h under identical conditions and further incubated with 280 µL kanamycin (50 mg/mL, final concentration) overnight. Next, this culture was centrifuged; the supernatant was conserved and 8 g of polyethylene glycol-8000 (PEG 8000, P3515, Sigma-Aldrich, St. Louis, MO, USA) and 4 g of NaCl were added. To precipitate the phages, the mixture was stirred at 300 rpm for 5 min at 37 °C. Later, samples were centrifuged at maximum speed, and the supernatant was discarded. Then, the phage pellet was air-dried at room temperature for 10 min.

#### 4.1.2. Phage Display for vNAR Selection

For the isolation of phages binding to the EGFRvIII peptide (pEGFRvIII, RP20356, GenScript, Piscataway, NJ, USA), the phage display was performed with four rounds of selection. Then, pEGFRvIII was immobilized in an ELISA plate to incubate with the phage displaying the vNARs domains that would specifically recognize the peptide. The wells of the ELISA plate were covered with 0.5 µg of the peptide (antigen) in 50 µL 1X PBS, enveloped with a plate sealer and incubated O/N at 4 °C. The following day, the antigen solution was discarded. Then, the wells were blocked with 50 µL of 3% BSA (*w*/*v*) in 1X PBS and the plate was incubated for 1 h at 37 °C. Afterwards, the blocking solution was discarded and 50 µL of the freshly prepared vNARs phage library was added to each well. Next, the plate was incubated for 2 h at 37 °C. Simultaneously, in a 15 mL sterile tube, 2 mL of SB medium was inoculated with 2 µL of electrocompetent *E. coli* TG1 and left to incubate with shaking at 250 rpm, for 1.5–2.5 h up to an OD of 1, and read at 600 nm for phage input titration (Input). The panning was continued, the phage solution was discarded to perform the astringent washes with 150 µL of 0.05% Tween20-1X PBS, pipetting up and down, and was followed by 5 min of incubation between each wash and then discarding that solution. The astringent washes were repeated 7 times in the first round, 14 times in the second round, 21 times in the third round, and 25 times in the fourth round (R0–R4). After washing, 50 µL of trypsin [10 µg/mL], freshly prepared in 1X PBS, was added to each well, incubating the plate at 37 °C for 30 min. Then, by vigorously pipetting 10 times up and down, the elution was transferred to the 2 mL *E. coli* culture. For the first round, 100 µL of elution per culture was used (2 wells each with 50 µL of elution). For subsequent rounds, 50 µL (1 well with 50 µL) was left to room temperature incubation for 15 min. Next, 6 mL of SB medium + 1.6 µL of ampicillin [100 µg/mL] were added to the culture by transferring the culture to a 50 mL polypropylene tube. Afterwards, the tube was stirred at 37 °C for 1 h at 250 rpm. Subsequently, 12 µL of ampicillin [100 µg/mL] was added and incubated at 37 °C for 1 h at 250 rpm. Output titrations were performed, diluting 2 µL of the culture in 198 µL of SB medium. Then, 100 µL and 10 µL of this dilution were seeded on plates with LB + ampicillin [100 µg/mL]. For input titrations, 50 µL of the prepared 2 mL *E. coli* culture was infected with 1 µL of a 10^−8^ dilution of the phage preparation, incubated for 15 min at room temperature, and plated on LB + ampicillin [100 µg/mL]. Once input titrations were plated, they were incubated O/N; then, the entry and exit of each round was calculated by multiplying the number of colonies for the culture volume and dividing the product by the plating volume. To the tube culture, 1 mL of M13K07 helper phage (10^13^ PFU, 18311-019, Thermo Scientific, Waltham, MA, USA) was added. The mixture in that tube was transferred to a 500 mL flask (sterile). This was followed by the addition of 91 mL of SB medium and 46 µL of ampicillin [100 µg/mL] to further incubate at 37 °C for 2 h at 300 rpm. Finally, 140 µL of kanamycin [50 µg/mL] was added and incubated at 37 °C and 300 rpm. As mentioned, one well was covered with theantigen and incubated O/N at 4 °C to prepare the plate for the next round. The culture was centrifuged at 4000 rpm for 15 min at 4 °C the following day. Next, the supernatant was transferred to a clean and sterile 500 mL flask. After that, 4 g of PEG-8000 (4% [*w*/*v*]) and 3 g of NaCl (3% [*w*/*v*]) were added, and the flask was left stirring at 300 rpm at 37 °C for 5 min. Subsequently, it was placed on ice for 30 min. At this time, the covering solution from the 96-well plate was incubated overnight before it was discarded and blocked with 150 µL of 3% BSA. Then, it was incubated at 37 °C for 1 h. Then, the culture was transferred to clean 50 mL tubes and centrifuged at 4500 rpm for 30 min at 4 °C. The supernatant was discarded, and the tubes were allowed to drain upside down on paper. Afterwards, the pellet in the tubes was carefully resuspended with 2 mL of 1% BSA and the volume was transferred to 2 mL sterile tubes. The 2 mL tubes were centrifuged at 13,000 rpm at 4 °C for 5 min, and then the supernatant was carefully taken and passed through a 0.22 µm syringe filter (GSWP04700, Merck Millipore, Burlington, MA, USA), leaving the phages in the filtered supernatant to continue with the next panning round.

#### 4.1.3. Colony PCR

Isolated colonies were analyzed by PCR and positive clones were sequenced, as previously described [39]. The presence of the vNAR-encoding gene presence in colonies from the R4 output was confirmed by colony PCR. Positive colonies with the expected size (350 bp) were cultured overnight with LB and ampicillin at 250 rpm and 37 °C. Then, plasmids were isolated with a PureYield Plasmid Miniprep System (A1223, Promega, Madison, WI, USA) kit, according to the supplier’s instructions. Plasmid integrity was assessed with SYBR Safe (S33102, Thermo Fisher Scientific, Waltham, MA, USA) agarose gel.

#### 4.1.4. Sequencing and In Silico Analysis of Coding Sequences

Sequencing of the plasmids from positive colonies was performed according to the conditions of Macrogen Inc. (Seoul, South Korea), or as directed by the LANBAMA laboratory (SLP, Mexico). To confirm the presence and integrity of vNAR genes, a multiple sequence alignment (MSA) with computational tools, Multalin interface [86] and ClustalX 2.1 [87], was performed after sequencing results. The reference sequence of vNAR was used with GenBank, AAX10146.1 access code.

#### 4.1.5. vNAR R426 Subcloning into pET28a+ Plasmid

To transfer the gene encoding the vNAR R426 from the initial pCOMb3X plasmid to the pET28a+ plasmid, a rigorous procedure of subcloning was followed. The vNAR R426 gene was amplified via PCR using vector-specific oligonucleotides with the GoTaq enzyme (M300, Promega), according to the manufacturer’s protocol. Both the amplicon and plasmid were digested with *NcoI-HF* and *XhoI-HF* (R0193 and R0146S, respectively, New England Biolabs, Ipswich, MA, USA), following the supplier’s conditions. Digestion enzymes were deactivated after digestion, and the digested amplicon was purified with the Monarch PCR & DNA Cleanup kit (T1030, New England Biolabs). Subsequently, the ligation of the amplicon and digested plasmid (3:1 ratio) was achieved with T4 ligase (M1801, Promega), and *E. coli* TOP10 was electroporated. The vNAR coding gene presence was confirmed with colony PCR using T7 oligonucleotides specific to the plasmids (T7 Forward 5′-TAATACGACTCACTATAGGG-3′; T7 Reverse 5′-GCTAGTTATTGCTCAGCGG-3′) and analyzed in a 2% agarose gel. Successfully transformed cells were cultured overnight in LB and ampicillin (100 μg/mL) at 250 rpm and 37 °C. Subsequent plasmid extraction was performed with the Monarch Plasmid Miniprep kit (T1110, New England Biolabs). The vNAR R426 plasmid was extracted and further sequenced by the LANBAMA laboratory. The alignment analysis was performed with MultAlin interface software version 5.4.1. Thus, the presence of the anti-EGFRvIII vNAR R426 coding gene within the plasmids was confirmed.

### 4.2. vNAR R426 Protein Expression

The pET28a+/vNAR R426 plasmid was employed to obtain electroporated and expression cultures of *E. coli* BL21 (DE3). Plasmid pET28a+ confers resistance to kanamycin [50 μg/mL final concentration]. The expression process was performed in a batch of 250 mL in 1 L flasks with the 2XYT culture medium, starting from a fresh plate (no more than a month of storage at 4 °C) and placed in a liquid medium with antibiotics to incubate overnight at 37 °C and 250 rpm. A 1:50 dilution of the pre-inoculum was used in the flasks with 250 mL of culture medium with kanamycin [50 μg/mL final concentration], which was allowed to grow at 37 °C and at 250–300 rpm until reaching an optical density (OD_600nm_) of ~0.5. Subsequently, after 2 h of incubation, the inducer IPTG [1 mM final concentration] was added. The induction temperature was maintained at 37 °C and stirring was increased to 300 rpm for 5 h. After induction, the culture was transferred to 50 mL tubes and centrifuged at 10,000 rpm for 5–10 min. The culture medium was discarded and the pellets were washed with sterile distilled water, centrifuged under the same conditions, and drained by turning the tube on blotting paper for 10 min. The tubes were labeled and frozen (−20 °C) for later protein extraction.

### 4.3. vNAR R426 Protein Extraction, Purification, and Quantification

The extraction and purification of the expressed vNAR R426 were carried out in a denaturing condition. For extraction, the pellet obtained from 200 mL of the culture was resuspended in 5 mL of sonication buffer (100 mM NaH_2_PO_4_, 10 mM Tris base pH 8), the cells were lysed by sonication with pulses at 500–600 W for 10 s, 4 times, with a rest period of 40 s between each pulse, followed by centrifugation at 10,000 rpm for 15 min at 4 °C. This was performed twice. The pellet was then resuspended in 4 mL of denaturing buffer B (100 mM NaH_2_PO_4_, 10 mM Tris base, 8 M urea pH 8). The tube was placed under agitation at 120 rpm on an orbital shaker for 90 min and centrifuged at 10,000 rpm for 20 min at 20 °C. Finally, the supernatant was transferred to new tubes and incubated with shaking at 120 rpm for 90 min with reduced glutathione (GSH) at a final concentration of 60 mM. The supernatant was added to 320 mL of renaturing buffer (50 mM Tris base, 5% *v*/*v* glycerol, 0.5 mM oxidized glutathione (GSSG), pH 8) and incubated with magnetic shaking for 16 h at 4 °C.

The vNAR R426 protein purification was carried out with metal affinity chromatography with the HisTrap HP column (5 mL) using the FPLC (Fast Protein Liquid Chromatography) equipment (GE healthcare ÄKTA™ Pure, Cytiva, Marlborough, MA, USA), starting from the initial volume of 320 mL of the renaturating buffer. The equipment was programmed with a flow rate of 2 mL/min to pass the sample and for elution acquisitions. The washing steps had a flow of 5 mL/min. The wash step (W1) was performed with the wash buffer 1 (50 mM NaH_2_PO_4_, 300 mM NaCl, 70 mM imidazole, pH 8). A single imidazole concentration gradient was used for the elution with an elution buffer (50 mM NaH_2_PO_4_, 300 mM NaCl, 300 mM imidazole, pH 8). The elution volume obtained from each elution (15 mL) was then dialyzed (68100, SnakeSkin Dialysis Tubing, 10 kDa molecular weight cut-off, Thermo Fisher Scientific) against two changes of 0.5X PBS and a final change of 0.5X PBS in a volume 400 times larger than the sample to be dialyzed. The dialysis procedure was performed at room temperature (R.T.), 200 rpm on a stirring plate, with changes every 2 h. Then, the last change was left stirring at 4 °C for 16 h. Subsequently, the analysis was performed on an SDS-PAGE gel (4% concentrator gel; 12% separator gel) and the analysis of purified vNAR R426 was performed. Further validation by Western blot analysis was achieved using an anti-HA-HRP antibody (ab1190, Abcam, Cambridge, UK) at a 1:5000 dilution. The concentration of each purified protein obtained was quantified after dialysis using the Micro BCA Protein Assay Kit (23235, Thermo Fisher Scientific) in an ELISA microplate following the protocol described by the manufacturer and read with the xMark plate reader (10013301X, Bio-Rad, Hercules, CA, USA).

### 4.4. Recognition ELISA

The anti-EGFRvIII recognition of the subcloned vNAR towards the EGFRvIII peptide was evaluated by a recognition ELISA. Then, 1 µg of pEGFRvIII was immobilized on an ELISA plate (triplicate) and incubated overnight at 4 °C. Afterwards, this solution was discarded and blocked with 3% BSA-1X PBS. Then, the purified vNAR R426 (1 µg) was deposited in the well. The detection of the vNAR R426 on the dilution was achieved with the anti-His-HRP antibody (MBS355032, MyBioSource, San Diego, CA, USA), and revealed with the 1-Step ultra TMB-ELISA substrate solution (34028, Thermo Scientific, Waltham, MA, USA), following the supplier’s instructions. Finally, the plate was read at 450 nm in the microplate reader xMark^TM^ (168–1150, Bio-Rad).

### 4.5. Characterization of the Interaction Between vNAR R426 and EGFRvIII Peptide by Molecular Docking and Molecular Dynamics

The structure of vNAR R426 was modeled using AlphaFold [88,89] and prepared for molecular dynamics (MD) simulations in Visual Molecular Dynamics (VMD) [90]. Simulations were executed with NAMD 2.14 [91], and the system was solvated in a water box with a 12 Å padding using the TIP3P water model and 0.15 M Na^+^ and Cl^−^ ions. Further analyses are detailed in Supplementary data. To determine the optimal starting position and orientation of pEGFRvIII relative to vNAR R426, predictions from the Hpepdock web server were employed [92]. The server selects the best docking score by evaluating binding poses based on a scoring function that estimates the binding free energy, incorporating van der Waals, electrostatic, and desolvation interactions. The pose with the most favorable (lowest) energy score, indicating the strongest binding affinity, is chosen as the best model, and was prepared for molecular dynamics (MD), and simulated with NAMD 2.14 using the CHARMM36 force field; the Root Mean Square Deviation (RMSD) and Root Mean Square Fluctuation (RMSF) were again calculated post-simulation. The binding free energy estimation for the vNAR R426-pEGFRvIII complex is depicted in Table S1.

### 4.6. Fluorescein Isothiocyanate (FITC)-Labeled Anti-EGFRvIII vNAR R426

The anti-EGFRvIII vNAR R426 was FITC-labeled (vNAR_FITC_) via EDC conjugation. Briefly, a conjugation buffer (0.1 M MES, pH 4.5–5.0) was employed with the EDC conjugation solution (1 mg/mL in DMSO). Next, vNAR R426 (500 µg) was dissolved in a conjugation buffer (500 µL). Next, 100 µL of EDC solution and 0.5 mg of FITC (Ex 494 nm; Em 518) were mixed with the dissolved vNAR R426 solution and incubated at room temperature for 2 h and then dialyzed in PBS 0.5X pH 3.5-4. Finally, the removal of endotoxins and vNAR_FITC_ concentration was performed with the ProteoSpin (22800, Norgen Biotek, Thorold, ON, Canada) kit, according to the manufacturer’s instructions.

### 4.7. vNAR_FITC_ Vesicular Transport Analysis

HBEC-5i and U87MG cells were seeded and cultured onto the Chamber Slide (130672, Thermo Scientific). The HBEC-5i (CRL-3245) and U87MG cells (HTB-14) were purchased from ATCC (Manassas, VA, USA). Each well contained 6 × 10^4^ cells (duplicate). The corresponding medium for the U87MG and HBEC-5i cell lines was employed according to the ATCC recommendations. Both cell lines were maintained at 37 °C and 5% CO_2_.

#### 4.7.1. Cytochalasin B

To evaluate the vNAR_FITC_ (0.081 µM/well) binding to EGFRvIII and internalization, HBEC-5i cells and U87MG were seeded and incubated with vNAR_FITC_ for 4 h.

The U87MG were treated with cytochalasin B (0.5 nM and 1 nM, C6762, Sigma Aldrich, St. Louis, MO, USA) for 4 h. Then, cells were fixed with 4% of paraformaldehyde for 30 min and permeabilized with 50% of ethanol for 1 min. The nucleus was further stained with a propidium iodide (PI, P4170, Sigma Aldrich) solution at a final concentration of 3 μM. After staining, vNAR_FITC_ was recognized via the fluorescence emission of FITC using a Leica DM5500 Confocal microscope (Leica Microsystem, Wetzlar, Germany) with the emission and excitation spectra for PI (Ex 535 nm; Em 617) and FITC (Ex 494 nm; Em 518) staining. The wells with HBEC-5i cells were used as a negative control because these are EGFR^−^/EGFRvIII^−^. The wells with U87MG cells were EGFRvIII^+^ and a positive control group was employed without cytochalasin B treatments. All experiments were conducted in triplicate.

#### 4.7.2. Brefeldin-A

To characterize the mechanistic basis of vNAR R426 binding and nuclear translocation along with EGFRvIII, U87-MG cells were seeded and cultured onto the Chamber Slide. Each well contained 6 × 10^4^ cells (duplicate). Then, the cells were treated with brefeldin-A (BFA, B7651, Sigma Aldrich) for 30 min (0.36 µM and 0.72 µM) and incubated with vNAR_FITC_ (0.081 µM/well) for 24 h. Finally, cells were fixed with paraformaldehyde 4% for 30 min and permeabilized with ethanol 50% for 1 min. The nucleus was further stained with propidium iodide (PI). After staining, vNAR_FITC_ was recognized via the fluorescence emission of FITC using a Leica DM5500 Confocal microscope using the same conditions as previously described.

### 4.8. Cell Viability Assays

In all cell viability assays were performed following the alamarBlue cell viability assay protocol. U87-MG cells were seeded in a 96-well plate. Each well contained 1 × 10^4^ cells in triplicate, incubated for 24 h under standard conditions. Briefly, once the experimental treatments were completed, the medium was discarded. Then, the 96-well plate was incubated with 100 µL of alamarBlue solution (1X) (DAL1025, Thermo Scientific) for 4 h. Following this incubation, the 96-well plate was measured at 570 nm and 600 nm. The negative control consisted of cells (without vNAR nor cisplatin) and the positive control consisted of cells treated with DMSO 20%. All experiments were conducted in triplicate.

#### 4.8.1. Cell Viability Cisplatin Assay in U87-MG Cell Line

After the 24 h of incubation, U87-MG cells (1 × 10^4^ cells/well in triplicate) were treated with different concentrations of cisplatin (1 µM, 5 µM, 10 µM, 50 µM, and 100 µM) for 72 h. Once the experimental treatments were completed, the medium was discarded. Then, the 96-well plate was incubated with 100 µL of alamarBlue solution (1X) for 4 h. Following this incubation, the 96-well plate was measured at 570 nm and 600 nm. The negative control consisted of cells (without vNAR nor cisplatin) and the positive control consisted of cells treated with DMSO 20%.

#### 4.8.2. Cell Viability Assay with vNAR 426 Alone

After the 24 h of incubation, U87-MG cells were (1 × 10^4^ cells/well) treated with different concentrations of vNAR R426 alone (0.024 µM, 0.049 µM, 0.097 µM, 0.19 µM, 0.49 µM, and 0.97 µM) for 48 h and 72 h. Once the experimental treatments were completed, the medium was discarded. Then, the 96-well plate was incubated with 100 µL of alamarBlue solution (1X) for 4 h. Following this incubation, the 96-well plate was measured at 570 nm and 600 nm. The negative control consisted of cells (without vNAR nor cisplatin) and the positive control consisted of cells treated with DMSO 20%. All experiments were conducted in triplicate.

### 4.9. vNAR R426 Conjugation with Cisplatin

The vNAR R426-CDDP (vNAR_CDDP_) coupling, with an approximate molar ratio of 1:1, was performed via EDC conjugation in the same conditions as FITC-labeled vNAR R426. Briefly, conjugation buffer (0.1 M MES, pH 4.5–5.0) was employed along with the EDC conjugation solution (1 mg/mL in DMSO). Next, 500 µg of vNAR R426 was dissolved in 500 µL of conjugation buffer. Shortly after, 100 µL of EDC solution and 500 µg/mL CDDP solution were mixed with the dissolved vNAR R426 solution and incubated at room temperature for 2 h. Subsequently, the solution was dialyzed (68100, Thermo Fisher Scientific) in PBS 0.5X pH 3.5–4. Finally, the removal of endotoxins and vNAR R426 protein concentration was performed with the ProteoSpin kit according to the manufacturer’s instructions. We confirmed vNAR_CDDP_ conjugation through absorbance in the Nanodrop equipment (Figure S1).

### 4.10. Cell Viability Assay with Cisplatin-vNAR R426 Carrier

U87-MG cells were seeded in a 96-well plate. Each well contained 1 × 10^4^ cells in triplicate and was incubated for 24 h. Thereafter, cells were incubated with different concentrations of vNAR_CDDP_ (0.024 µM, 0.049 µM, 0.097 µM, 0.19 µM, 0.49 µM, and 0.97 µM) for 48 h and 72 h. Once the experimental treatments were completed, the medium was discarded. Then, the 96-well plate was incubated with 100 µL of alamarBlue solution (1X) for 4 h. Following this incubation, the 96-well plate was measured at 570 nm and 600 nm. The negative control consisted of cells (without vNAR nor cisplatin) and the positive control consisted of cells treated with DMSO 20%.

### 4.11. Statistics

The statistical analysis was assessed by One-way ANOVA between different groups and Student’s *t*-test with a significance level set at *p* < 0.05 in the Origin Pro8^®^ and GraphPad prism 10 software.

## 5. Conclusions

In the present study, we report for the first time the successful isolation, characterization, and evaluation of the first single domain anti-EGFRvIII vNAR from a non-immune freshwater stingray mixed library. To the best of our knowledge, the isolation of vNARs of a non-immune library from freshwater stingrays has not been reported previously. This could represent an alternative and low-cost source for vNAR isolation concerning their marine counterparts. Our panning of a non-immune mixed library resulted in the selection of the vNAR domain named R426, which demonstrated the recognition of EGFRvIII in the in vitro and in silico analyses. We characterized the interaction of vNAR and pEGFRvIII by molecular docking and molecular dynamics. The molecular dynamics results demonstrated the conformational stability of vNAR R426 in complex with pEGFRvIII. The subsequent conjugation of vNAR R426 with FITC was performed to evaluate the vNAR R426 in vitro internalization by cell immunofluorescence staining and confocal microscopy analysis. We also employed cytochalasin B, a vesicle formation inhibitor, to confirm vNAR R426 internalization by receptor-mediated endocytosis. These results suggested that vNAR R426 enters the cells via receptor-mediated endocytosis, as the FITC-labeled-vNAR R426 (vNAR_FITC_) signal was only detected in U87-MG cells (EGFRvIII^+^). On the other hand, no signal was detected in HBEC-5i cells (native EGFR^−^/EGFRvIII^−^). These findings also implied that vNAR R426 could be internalized and found in the nucleus of U87-MG. To further validate this retrograde trafficking and nuclear translocation of vNAR R426 alongside EGFRvIII by a previously reported COPI-mediated EGFRvIII nuclear translocation mechanism [50], we treated U87-MG with brefeldin-A (BFA), an inhibitor of the shuttle function of COPI vesicles. Thereafter, we successfully detected the vNAR_FITC_ signal within the control groups, whereas no vNAR_FITC_ signal was detected in the BFA-treated U87-MG cells. These findings implied that vNAR_FITC_ nuclear translocation relied on EGFRvIII nuclear translocation mediated by COPI, establishing the mechanistic basis for vNAR R426, and the inhibition of this mechanism also hampers the vNAR_FITC_ signal within the nucleus. Based on this assumption, we conjugated vNAR R426 with CDDP (vNAR_CDDP_) to evaluate the targeted delivery of cisplatin by EGFRvIII-mediated endocytosis in U87-MG cells. These results proved highly significant differences between the cell survival of the maximum concentration of vNAR_CDDP_ (0.97 µM) when compared to free CDDP (10 µM). The IC_50_ of cisplatin was remarkably improved by at least one order of magnitude owing to its conjugation to vNAR R426 (vNAR_CDDP_) when compared to free CDDP (10 µM). Notably, the required IC_50_ of the vNAR_CDDP_ (0.97 µM) is 10-fold smaller than the required IC_50_ of free CDDP (10 µM) to achieve greater efficacy in the U87-MG cells at 72 h (30% cells). We hypothesized that vNAR R426 internalization by receptor-mediated endocytosis, followed by the conjugation of CDDP and subsequent COPI-mediated EGFRvIII translocation to the nucleus, could induce the compelling cytotoxic effect observed in U87-MG cells when compared to free CDDP. Nevertheless, further validation of the molecular mechanisms and signaling pathways is required to verify this hypothesis.

Moreover, we also reviewed the potential application of vNARs for glioblastoma-targeted therapy via receptor-mediated endocytosis in detail and put them forward to develop vNARs as therapeutic carriers of antitumor drugs for GBM-targeted therapy [93]. In this study, we report the isolation and characterization of the first antigen-specific EGFRvIII vNAR (vNAR R426), described the vNAR R426 internalization mechanism by receptor-mediated endocytosis and the subsequent COPI-mediated nuclear translocation of EGFRvIII, and highlighted the importance of this shuttle mechanism to enhance the targeted delivery of cisplatin within the glioma cell’s nucleus and improve cytotoxic effects. In conclusion, vNAR R426 could be a potential therapeutic carrier for EGFRvIII-targeted glioblastoma and EGFRvIII-based cancer therapies.

## Data Availability

Data will be made available on request.

## Supplementary Materials

The following supporting information can be downloaded at: https://www.mdpi.com/article/10.3390/ijms26030876/s1. References [94,95,96,97,98] are cited in the Supplementary Materials.
