# Supplementary material for: Isolation and Characterization of the First Antigen-Specific EGFRvIII vNAR from Freshwater Stingray (Potamotrygon spp.) as a Drug Carrier in Glioblastoma Cancer Cells
Source: Int J Mol Sci. 2025 Jan 21;26(3):876. doi: 10.3390/ijms26030876 (PMC11817625; doi:10.3390/ijms26030876)
Supplement: Supplementary file 1 [file ijms-26-00876-s001.zip › ijms-3410504-supplementary.pdf]

Article

Isolation and Characterization of the First Antigen-Specific EGFRvIII vNAR from Freshwater Stingray (*Potamotrygon* spp.) as a Drug Carrier in Glioblastoma Cancer Cells

Alejandro Manzanares-Guzmán [1], Andrea C. Alfonseca-Ladrón de Guevara [1], Elia Reza-Escobar [1], Mirna Burciaga-Flores [2], Alejandro Canales-Aguirre [1], Hugo Esquivel-Solís [1], Pavel H. Lugo-Fabres [3] and Tanya A. Camacho-Villegas [1,*]

[1] Unidad de Biotecnología Médica y Farmacéutica, Centro de Investigación y Asistencia en Tecnología y Diseño del Estado de Jalisco (CIATEJ), Guadalajara C.P. 44270, Jalisco, Mexico; almanzanares_al@ciatej.edu.mx (A.M.-G.); analfonseca_al@ciatej.edu.mx (A.C.A.-L.d.G.); eliarezza@gmail.com (E.R.-E.); acanales@ciatej.mx (A.C.-A.); hesquivel@ciatej.mx (H.E.-S.); tcamacho@ciatej.mx (T.A.C.-V.)

[2] Centro de Nanociencias y Nanotecnología, Universidad Nacional Autónoma de México (CNyN-UNAM), Carretera Tijuana-Ensenada km107, Ensenada C.P. 22860, Baja California, Mexico; mirna.b.flores@ens.cnyn.unam.mx (M.B.F.)

[3] CONAHCYT-Unidad de Biotecnología Médica y Farmacéutica, Centro de Investigación y Asistencia en Tecnología y Diseño del Estado de Jalisco (CIATEJ), Guadalajara C.P. 44270, Jalisco, Mexico; plugo@ciatej.mx (P.H.L-F.)

* Correspondence: tcamacho@ciatej.mx

SUPPLEMENTARY DATA

1. Material and methods

1.1. Characterization of the interaction between vNAR R426 and EGFRvIII peptide by molecular docking and molecular dynamics

The structure of vNAR R426 was modeled using AlphaFold [88,89] and prepared for molecular dynamics (MD) simulations in Visual Molecular Dynamics (VMD) [90]. Simulations were executed with NAMD 2.14 [91], and the system was solvated in a water box with a 12 Å padding using the TIP3P water model and 0.15 M Na+ and Cl− ions. An initial minimization of 20,000 steps was performed using the conjugate gradient method, followed by a heating phase via simulated annealing, raising the temperature from 0 °K to 400 °K over 100 ps. The system was maintained at 400 °K for 100 ps, then cooled to 300 °K over 200 ps, and pre-equilibrated at 300 °K for an additional 600 ps. Following the heating phase, equilibration was conducted for 5 ns under constant particle number, temperature, and pressure (NPT). A subsequent production phase was run for 100 ns under NPT conditions. Post-simulation, Root Mean Square Deviation (RMSD) and Root Mean Square Fluctuation (RMSF) were calculated using the Python MDAnalysis library [94], with visualization and analysis carried out in VMD. The quality of vNAR R426 structures pre- and post-MD simulation was evaluated through Ramachandran plots generated using the MolProbity server (http://molprobity.biochem.duke.edu/) [95]. To determine the optimal starting position and orientation of pEGFRvIII relative to vNAR R426, predictions from the Hpepdock web server were employed [92]. The best-scoring complex was selected, prepared for MD, and simulated with NAMD 2.14 using the CHARMM36 force field. RMSD and RMSF were calculated as post-simulation.

1.2. Estimation of the binding free energy estimation vNAR R426-pEGFRvIII complex by MM-GBSA method.

Binding free energy for the vNAR R426-pEGFRvIII complex was estimated using the molecular mechanics generalized Born surface area (MM/GBSA) method, with the MM-PBSA.py plugin from AmberTools23 [96,97]. The CHARMM to AMBER force field

conversion was carried out using ParmED [98]. Three independent MD assays were performed for both the isolated vNAR R426 and its complex with pEGFRvIII.

2. Results

2.1. The binding free energy estimation by MM-GBSA method.

The Free Energy of Binding using the MM/GBSA method over 100 ns of MD for the vNAR R426 and EGFRvIII peptide complex indicates a favorable attraction between the molecules. The van der Waals energy (-35.376 kcal/mol) and electrostatic energy (-79.139 kcal/mol) suggest significant attractive forces. Although the polar solvation energy is positive (98.818 kcal/mol), the negative contribution from the non-polar surface energy (-5.547 kcal/mol) and gas phase interactions (ΔG gas = -114.518 kcal/mol) compensates for this effect. Overall, the negative total free energy (ΔG total = -21.247 kcal/mol) suggests stable binding of the complex under the simulated conditions, which could imply high affinity and stability of the vNAR R426-pEGFRvIII interaction in a biological environment.

Table S1. Binding free Energy using MM/GBSA method over 100 ns of molecular dynamics.

Energy Component	Average Kcal/mol
VDWAALS	-35.376
EEL	-79.139
EGB	98.818
ESURF	-5.547
ΔG gas	-114.518
ΔG solv	93.271
ΔG total	-21.247

Note: Different binding free energies values in kcal/mol calculated by MM-GBSA method: VDWAALS (van der Waals energy), EEL (electrostatic energy), EGB (MM/GBSA polar solvation energy), ESURF (MM/GBSA non-polar solvation energy), ΔG gas (net gas phase energy), ΔG solv (net solvation energy), ΔG total (net system energy). The results are an average of 3 experimental replicates. 2.2 Physicochemical properties of vNAR R426.

The vNAR-R426 cloned in pCOMB3X vector is composed of 136 amino acids (113 correspond to vNAR R426 and the additional amino acids residues belong to SfiI recognition site, His6x-Tag, and HA-Tag). The calculated molecular weight of vNAR-R426 was 14.95 kDa. The amino acid composition includes 15 negatively charged residues (Asp + Glu) and 10 positively charged residues (Arg + Lys), suggesting a charge distribution that can influence both solubility and stability. The extinction coefficient, measured at 280 nm in water, is 30,285 M^{-1} cm^{-1}, facilitating concentration estimation via absorbance. The protein's instability index of 29.29 indicates that it is stable, as values below 40 indicate stability. Its aliphatic index of 52.35 indicates the volume occupied by aliphatic amino acids, suggesting moderate thermal stability. Furthermore, the average hydropathicity (GRAVY) is -0.524 indicates slight hydrophilicity, which could enhance its solubility in aqueous media (Table S2).

Table S2. Physicochemical properties of the vNAR R426 domain computed by Protparam available in Expasy server.

**Physicochemical parameter**	**vNAR R426**
Number of amino acids:	136
Molecular weight: (KDa)	14.95
Theoretical pI:	5.23
Ext. coefficient	30285
Estimated half-life:	30 hours (mammalian reticulocytes, *in vitro*).<br>>20 hours (yeast, *in vivo*)<br>>10 hours (*Escherichia coli*, *in vivo*).
Instability index:	29.29
Aliphatic index:	52.35
Grand average of hydropathicity (GRAVY)	-0.489

*Note: Physicochemical properties were computed using R426 sequence (113 aa) plus SfiI recognition site, His6xTag and HA tag.

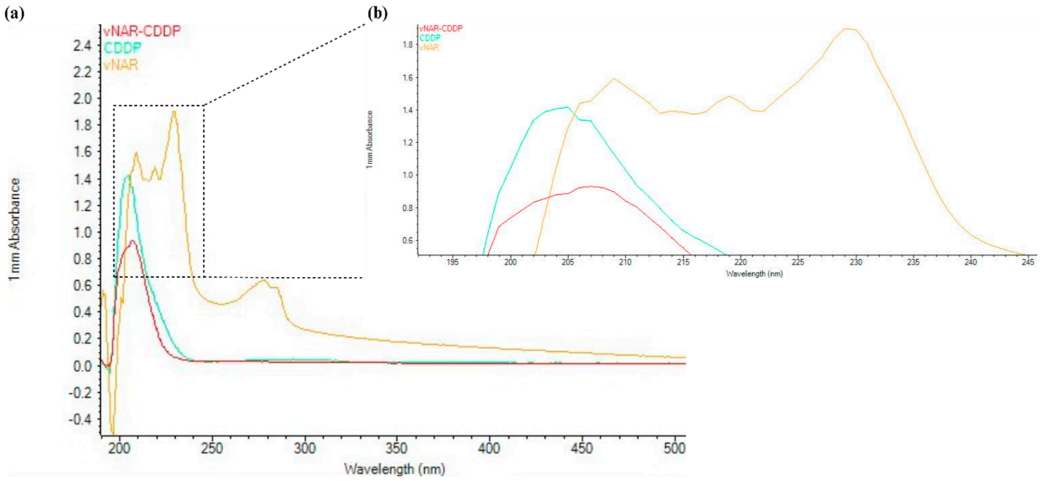

Figure S1. Absorbance of vNARCDDP conjugation in the Nanodrop equipment. (a) Absorbance of conjugated vNAR with CDDP (red), free CDDP (teal) and free vNAR (yellow) are depicted from 190-500 nm. (b) Zoomed section of the absorbance of vNARCDDP (red), free CDDP (teal) and free vNAR (yellow) from 195-245 nm.
